# Isolation of High Purity Anthocyanin Monomers from Red Cabbage with Recycling Preparative Liquid Chromatography and Their Photostability

**DOI:** 10.3390/molecules23050991

**Published:** 2018-04-24

**Authors:** Yijun Chen, Zikun Wang, Hanghang Zhang, Yuan Liu, Shuai Zhang, Qingyan Meng, Wenjie Liu

**Affiliations:** 1 Xinjiang Production & Construction Group, Key Laboratory of Biological Resource Protection and Utilization of Tarim Basin, Alar 843300, China; 18810619033@163.com (Y.C.); hang19930426@163.com (H.Z.); zsno702121@163.com (S.Z.); 2 School of Chinese Materia Medica, Beijing University of Chinese Medicine, Beijing 100102, China; 3 Analytic Center, Tarim University, Alar 843300, China; 15292501912@163.com; 4 Ethnic Medicine Institute, Southwest University for Nationalities, Chengdu 610041, China; yuanliu163@aliyun.com

**Keywords:** anthocyanins, red cabbage, recycling preparative high performance liquid chromatography, stability

## Abstract

Anthocyanins from red cabbage are of great importance for their applications in the food industry as natural colorants and their beneficial effects on human wellness as natural antioxidants. This study aimed to develop an effective method for the isolation of anthocyanins with the help of a combination of alternate recycling and direct recycling preparative liquid chromatography. Ten major components of anthocyanins from red cabbage were isolated and their structures were identified by HPLC-MS/MS. Meanwhile, the stability of the isolated anthocyanins under various light conditions was also investigated so as to provide data for their storage. In sum, the results showed that twin column recycling preparative chromatography is an effective method for the isolation of anthocyanin monomers with similar structures. Besides, the stability of various anthocyanins from red cabbage was related to the number of acylated groups and mainly affected by illumination.

## 1. Introduction

Red cabbage (*Brassica oleracea* L.) is one of the most recognized healthy vegetables belonging to the Brassicaceae family that is grown and eaten worldwide for its various nutrition, such as vitamins, inorganic elements, beta-carotene, protein, and so on [1,2]. Meanwhile, red cabbage is also best known for its quantities of anthocyanins [3]. Interestingly, the color of the anthocyanins in red cabbage varies from red at low pH to blue and green at high pH [4], different from that of grape skins, black currants, and elderberries [5], thus making it popular as a natural colorant in the food industry. Previous research has shown that the anthocyanins have rich pharmacological activities, for instance: antioxidant [6], antihyperlipidemia [7,8], cardiovascular protecting [9,10], liver protection [11], and so on [12,13,14]. Besides, anthocyanins are becoming more and more popular throughout the world instead of synthetic pigments [15]. Additionally, it could also be concluded that the anthocyanins are of considerable research interest for human wellness.

**Figure 1:**
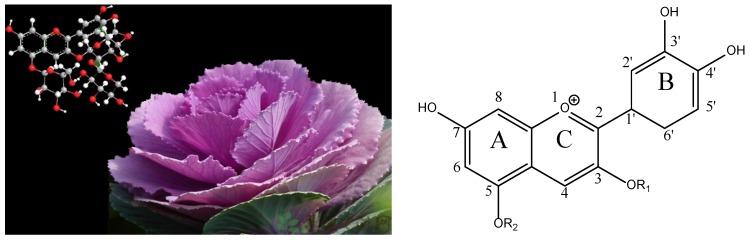
A color illustration of an identified anthocyanin coupled with red cabbage and the chemical structure of an anthocyanin mother nucleus in red cabbage.

**Figure 2:**
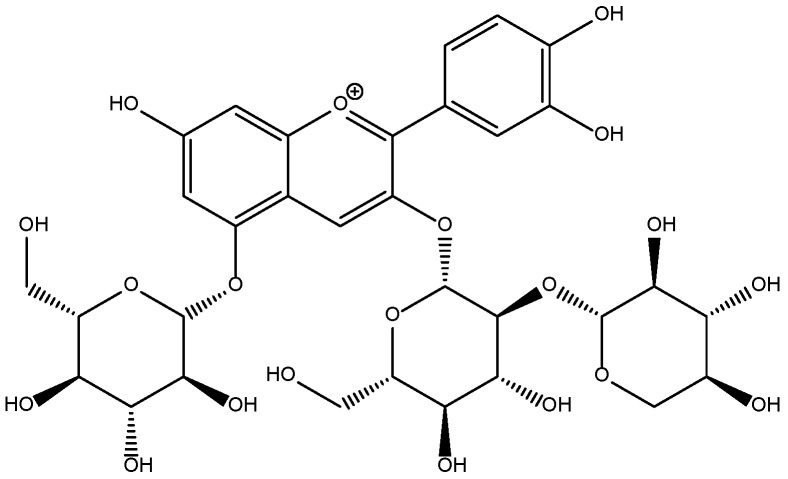
The identified chemical structure of an anthocyanin in red cabbage (Cy-3-soph-5-Glc).

It has been reported that anthocyanins are glycosylated polyhydroxy and polymethoxy derivatives of flavylium salts with electron-deficient chemical structures (Figure 1), which makes them easy to react with reactive oxygen [16,17]. The diversity of anthocyanins depends on the number and position of glycosides attached to the aglycone that can be acylated with various acylation groups. Furthermore, the main anthocyanins in red cabbage are derivatives of cyanidin (Figure 2) that are highly acylated with different numbers of cinnamyl or benzoyl groups [18]. High purity anthocyanin standards are essential for the quantitative and qualitative analysis of anthocyanins from various fruits and vegetables. However, it is easy to change the structures of high purity anthocyanins exposed to light, heat, oxygen, and other factors, making it difficult to separate the anthocyanins using traditional separation methods [19,20].

It is well known that preparative high performance liquid chromatography (p-HPLC) is an efficient and reliable approach to separate natural compounds [21]. However, it also has a deficiency in terms of the isolation of compounds with similar structures. Commonly used recycling preparative chromatography could achieve a higher separation power by prolonging the length of the column [22,23]. However, this method also has some shortcomings, such as: waste of solvents, chromatographic peak extension, decrease in production, and so on. So, in this study, based on previous research, we designed a versatile method (Figure 7) using a combination of alternate recycling and direct recycling preparative for the isolation of high purity anthocyanin monomers that have a similar structure and even similar retention times in red cabbage. In addition, the isolated anthocyanin monomers were further investigated under various conditions and the association between their structure and stability was then discussed. 

## 2. Results 

### 2.1. HPLC Analysis of Red Cabbage Anthocyanins and Preparative Scale Isolation

**Figure 3:**
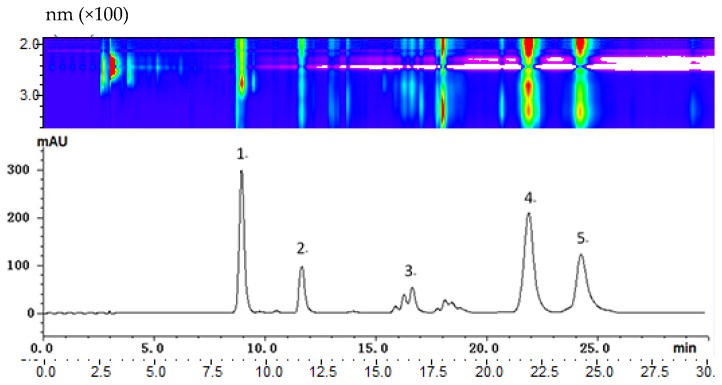
The HPLC chromatogram of the red cabbage extract was recorded at 520 nm, and the two-dimensional spectra were covered at 200–800 nm.

The purpose of our study was to determine the feasibility of a preliminary pilot separation for large-scale preparative HPLC isolation. This was done by implementing a small scale separation on an analytical column and directly increasing the preparative scale. With our HPLC conditions, the maximum absorption wavelengths of most anthocyanin peaks were approximately 520 nm, and thus, this value was selected for the demonstration of the HPLC chromatograms. Figure 3 shows an analytical HPLC chromatogram at 520 nm for the separation of total crude red cabbage anthocyanins. Because there are clearly four major peaks in the chromatogram with sufficient resolution between them, we increased the scale to preparative separation by loading a 100 mg crude sample. For each preparative isolation, a 2 mL 50 mg·mL^−1^ sample was injected, and the fractions were collected with an automatic fraction collector. The preparative separation was repeated five times, and the same fractions were combined for purity analysis.

Five fractions were obtained from the first preparative isolation and were evaluated with HPLC-MS/MS analysis for purity. Among them, fraction 1 and fraction 2 were obtained with a sufficient purity of 97.7% and 98.2%, respectively. Fraction 3 appeared as a broad peak in the preparative chromatogram and showed three major components in the HPLC-MS/MS analysis. Although a single peak was obtained for fraction 3, HPLC-MS/MS showed that there were three major anthocyanins that were unresolved. Similarly, fraction 4 showed two major constituents, and fraction 5 showed three major constituents that were unresolved and subjected to further recycling isolation.

### 2.2. Isolation of Anthocyanin Monomers with Recycling Preparative Chromatography

**Figure 4:**
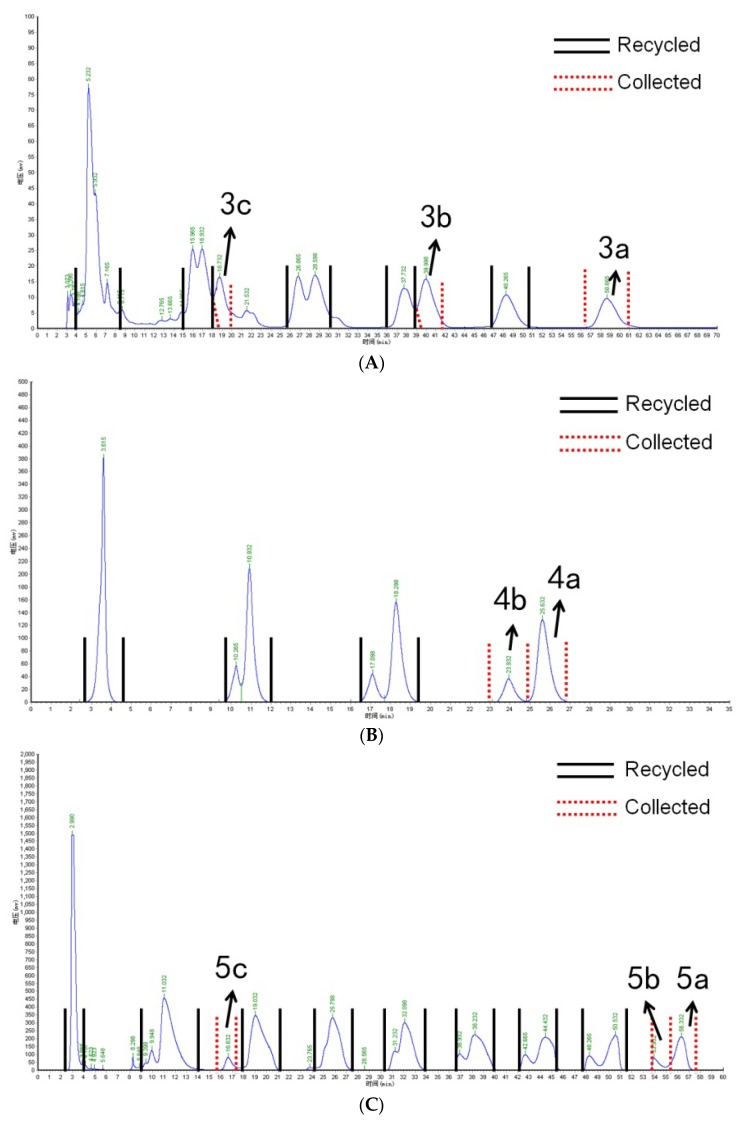
(**A**–**C**) respectively represent the recycling preparative HPLC chromatogram of fractions 3, 4, and 5.

**Figure 5:**
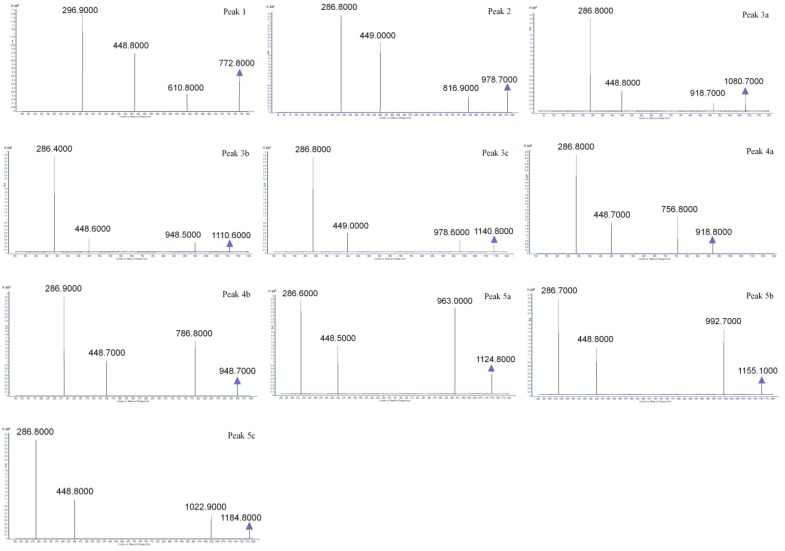
The ESI-MS2 spectra of ten monomeric anthocyanins in red cabbage. The MS was operated in positive mode. “
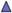
” represents the parent ion peak for each anthocyanin ([M + H]^+^).

Preliminarily separated fractions 3, 4, and 5 were further isolated using recycling preparative chromatography. For fraction 3, isocratic elution was performed with 30:70 methanol:water with 3% formic acid, and the result is shown in Figure 4A. The recycling isolation of fraction 4 is shown in Figure 4B, and the isolation results for fraction 5 are shown in Figure 4C. Meanwhile, the MS2 spectra of the isolated anthocyanin monomer is shown in Figure 5. 

For the isolation of Cy-3-(caff-pC)-diGlc-5-Glc, Cy-3-(glucofer)-diGlc-5-Glc, and Cy-3-(glucosin)-diGlc-5-Glc, 2 mL of fraction 3 was injected into the recycling system. From the third cycle, compound 3c (Cy-3-(glucosin)-diGlc-5-Glc) was baseline separated from the other two components and thus collected. However, anthocyanins 3b and 3a showed little separation from the third cycle and were subjected to further recycling separation. After five cycles, 3b and 3a were completely resolved and thus separately collected. The amounts for 3a, 3b, and 3c were 4.2 mg, 10.7 mg, and 21.0 mg, respectively. Figure 4B,C provides the chromatogram of recycled preparative isolation for anthocyanins 4a, 4b and 5a, 5b, 5c, respectively.

A total of 10 anthocyanins were separated using preparative and recycling preparative chromatography, including Cy-3-soph-5-Glc, Cy-3(sin)-diGlc-5-Glc, Cy-3-(caff-pC)-diGlc-5-Glc, Cy-3-(glucofer)-diGlc-5-Glc, Cy-3-(glucosin)-diGlc-5-Glc, Cy-3-(pC)-diGlc-5-Glc, Cy-3-(fer)-diGlc-5-Glc, Cy-3-(fer)(fer)-diGlc-5-Glc, Cy-3-(sin)(fer)-diGlc-5-Glc, and Cy-3-(sin)(sin)-diGlc-5-Glc, with purities of 97.7%, 98.2%, 95.3%, 96.4%, 98.1%, 96.9%, 99.2%, 97.5%, 98.1%, and 99.5%, respectively. The isolated compounds were identified using an analysis that included the HPLC-MS/MS data combined with UV-visible spectra and the elution order of peaks. The results of identification and MS/MS ions are summarized in Table 1. The identification of anthocyanidin was confirmed by the product ion value of 287, which is the *m*/*z* of the cyanidin aglycone. Of all isolated anthocyanins, their maximum absorption wavelengths were obtained with a PDA detector. The absorption wavelengths at approximately 330 nm indicate that the acylated group was present. Peak **1** showed no absorbance at approximately 330 nm, and this compound was identified as Cy-3-soph-5-Glc.

### 2.3. Photostability of Isolated Anthocyanins from Red Cabbage

**Figure 6:**
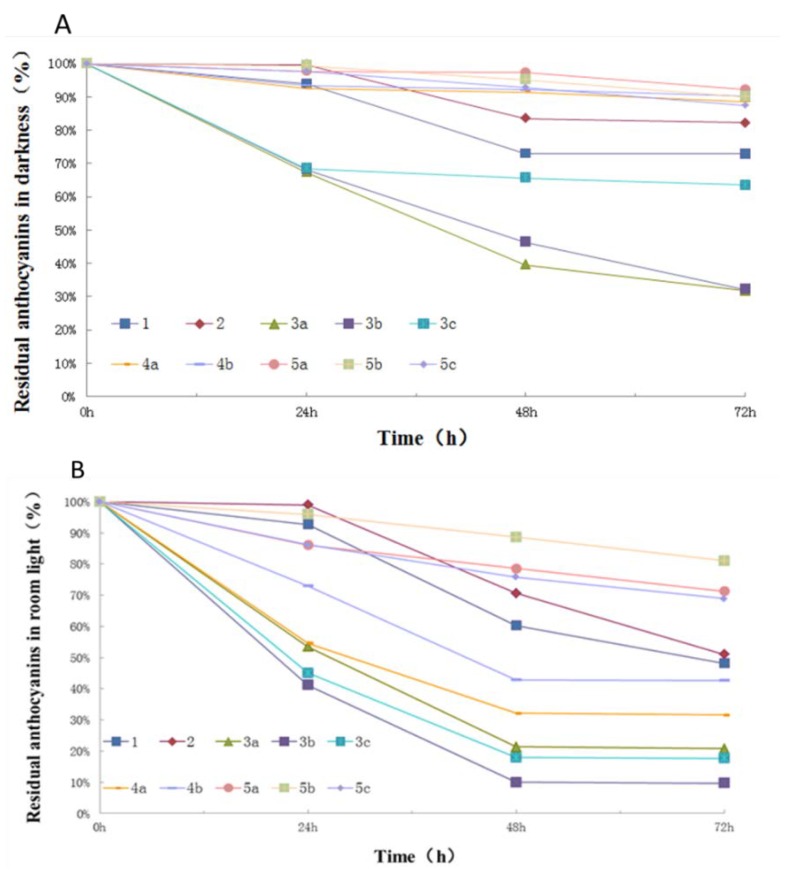

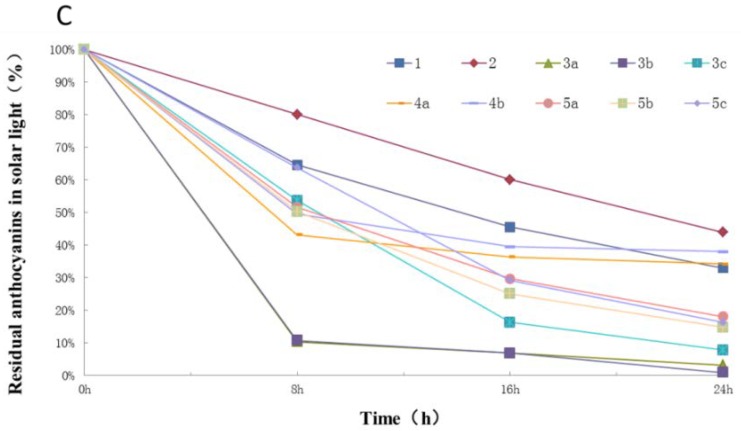
Stability of anthocyanins in darkness (**A**), exposed to room light (**B**) and simulated solar light (**C**).

As shown in Figure 6A–C, the degradation of individual anthocyanins appeared to show a similar trend in different light irradiation conditions but different degradation rates. In darkness, Cy-3(fer)-diGlc-5-Glc, Cy-3-(fer)(fer)-diGlc-5-Glc, and Cy-3-(sin)(fer)-diGlc-5-Glc decreased to 90, 92, and 90%, respectively, after 72 h at room temperature. Additionally, Cy-3-(caff-pC)-diGlc-5-Glc, Cy-3-(glucofer)-diGlc-5-Glc, and Cy-3-(glucosin)-diGlc-5-Glc decreased to 67%, 68%, and 68%, respectively, in 24 h. The concentration of Cy-3-soph-5-Glc and Cy-3(sin)-diGlc-5-Glc only decreased by 6% and 1%, respectively, in 24 h; however, they decreased rapidly to 73% and 83%, respectively, after 48 h and then maintained a relatively stable curve over the next 24 h. The degradation rate of various anthocyanins is related to the degree of acylation. In general, the more acyl groups that are attached to the anthocyanin, the faster the degradation speed that is observed in darkness.

Natural room light irradiation obviously increased the process of degradation of anthocyanin compared to the darkness experiments. After 72 h, Cy-3(fer)-diGlc-5-Glc, Cy-3-(fer)(fer)-diGlc-5-Glc, and Cy-3-(sin)(fer)-diGlc-5-Glc were decreased to 43%, 71%, and 81%, respectively. Cy-3-soph-5-Glc and Cy-3(sin)-diGlc-5-Glc were decreased to 93% and 99%, respectively, in 24 h, and then decreased to 60% and 71%, respectively, in 48 h, and they finally reduced to 48% and 51%, respectively, in 72 h.

All tested anthocyanins decomposed rapidly in the simulated solar light irradiation experiments, as shown in Figure 6C. After 24 h of irradiation, Cy-3-soph-5-Glc and Cy-3(sin)-diGlc-5-Glc decreased to 33% and 44%, respectively, and only a trace level of Cy-3-(glucofer)-diGlc-5-Glc remained after 24 h. These observed results are in agreement with previous reports regarding the photostability of anthocyanins under various conditions [31,32]. Furthermore, the photostability of red cabbage anthocyanin monomers is significantly affected by the number of acylated groups. Anthocyanins with more acyl groups appeared more labile to photodegradation.

## 3. Discussion

Recent research showed that anthocyanins richly concentrated in vegetables and fruits may have potential cancer prevention properties [33], as well as anti-aging [34], anti-inflammation [35], and anti-radiation [36] activities. Additionally, red cabbage is a major source of anthocyanins for the coloration of food due to its rich content and unique feature that it exhibits color over a very broad pH range. However, due to their unstable activities and similar chemical structure, just a few high purity anthocyanin monomers such as cyaniding-3-glucoside are sold on the market. Meanwhile, the price of them is also very high. To our knowledge, there were some papers reported before related to the separation of anthocyanins, for example: Yi [37] isolated three anthocyanins from red cabbage using high-speed counter-current chromatography (HSCCC) and the purities of them were 76.28%, 45.46%, and 91.46%, respectively; Yu [38] isolated one anthocyanin from blueberry with the help of medium pressure column chromatography on macroporous resin and sephades LH-20; Chen [39] isolated two anthocyanins from mulberry using five different types of macroporous absorbent resins; and so on. However, these methods are fraught with several disadvantages, in that they are time-consuming, laborious, expensive with poor recovery, and unsuitable for large-scale industrial production. 

The recycling p-HPLC has rapid and efficient advantages in the separation of natural compounds and some researches have been reported [40,41]. However, different from the papers reported before, this study developed a more effective and economical method with the help of the combination of alternate recycling and direct recycling preparative liquid chromatography. The two position ten-way valve was carried out to make the compounds continuously separate into two independent columns. Meanwhile, the flowing phase transitioned through the six-way valve to participate in the cycle. So, in this way, the complex samples could be efficiently separated in three approaches including alternate circulating, direct cycling, and a combination of the two. In contrast with the previous technique, the method we established has the characteristics of being flexible, solvent saving, and high purity, and has an efficient separation power, therefore being meaningful.

In addition, the results showed that only derivatives of cyanidin had been discovered in red cabbage pigment extract and these anthocyanins were highly acylated with different numbers of cinnamyl or benzoyl groups. In the meantime, the stability of anthocyanins in red cabbage was correlated with the number of acyl groups and was mainly affected by the illumination. Besides, the structures of high purity anthocyanin monomers were easy to change in a solution state. However, a low pH value was good for the stability of samples and the dry powder stored in darkness was supposed to be the best preservation method of anthocyanin monomers.

## 4. Materials and Methods 

### 4.1. Chemicals

HPLC grades of methanol, formic acid, and acetonitrile were purchased from Shanghai Anpel Scientific Instrument Co., Ltd., Shanghai, China (made by CNW Technologies GmbH, Dusseldorf, Germany). AR grades of ethanol, ethyl acetate, and hydrochloric acid were purchased from Beijing Chemistry Factory (Beijing, China). XDA-8 macro mesh resin was also purchased from Anpel Scientific Instrument Co., Ltd. (Shanghai, China). Deionised water was obtained from a Milli-Q Element water purification system (Millipore Corp., Billerica, MA, USA).

### 4.2. Sample Preparation

For the extraction of the total anthocyanins from red cabbage, 1.6 kg of fresh red cabbage was purchased from a local supermarket, cut into small pieces, and then homogenized with a blender. The homogenized sample was extracted with 3 liters of 50:50 methanol:water (*v*/*v*, with 1% formic acid added separately) in an ultrasonic bath for 1 hour at room temperature, and then filtered under vacuum. The residue was then extracted twice using the same procedure as that of the initial extraction. The filtrates were combined and concentrated in a rotary evaporator at 40 °C to approximately one third of their original volume to afford a dark red solution. The concentrated solution was then loaded onto a XDA-8 macro mesh resin column (50 × 3 cm) with a flow rate of 6 mL·min^−1^. All sample solutions were loaded onto the column, the column was eluted with a 3-bed volume of water with 1% formic acid to remove sugar and inorganic components, and the column was then eluted using 80% (*v*/*v*) methanol water with 3% formic acid. The colored eluent was concentrated in a rotary evaporator and then freeze-dried to afford a dark red powder of total crude anthocyanin extract weighing 2.048 g.

### 4.3. HPLC Analysis

The analytical chromatographic system consisted of an Agilent 1200 HPLC dual pump solvent delivery system with an auto sampler and a photodiode array detector that was connected to an Agilent 6410B quadrupole mass spectrometer by an electrospray ionization source. The analyses were performed with a Waters cortex C18 core-shell column (100 mm × 2.1 mm i.d., 2.6 µm). The separation was carried out under ambient temperature. Mobile phase A consisted of anhydrous formic acid and water (3:97 *v*/*v*), and mobile phase B consisted of anhydrous formic acid and methanol (3:97 *v*/*v*). The gradient program was optimized as follows: 0–15 min, 10% to 35% B; 15–35 min, 35% to 45% B; 35–45 min, 45% to 100% B; 45–55 min, 100% B. The equilibration time between runs was 12 min. The injection volume was 3 µL, the mobile phase flow was 0.2 mL·min^−1^, and the detection wavelength for the photodiode array (PDA) was 520 nm. For MS detection, electrospray ionization (ESI) was performed in the positive mode, the nebulizer pressure 30 psi, N2 drying gas 13 L·min^−1^, drying gas temperature 350 °C, capillary voltage 4000 V, and the mass scan range was from 100 to 1500 *m*/*z*.

### 4.4. Preparative HPLC

A Waters preparative high-performance liquid chromatography system equipped with a 2489 UV/visible detector, a 2545 binary gradient module, a 2767 sample manager, and a fraction collector was used for the isolation of anthocyanins from red cabbage extract, which was guided by the analytical HPLC results. The separation was performed with a Waters Sunfire C18 (19 × 250 mm, 10 μm) column using the same gradient elution as that which was used with the analytical HPLC methods. The flow rate was 15 mL·min^−1^, and the same mobile phase was used as that which was used for the analytical HPLC analysis. The sample solutions were prepared with 100 mg·mL^−1^ of crude anthocyanin. For each preparative injection, 50 mg of total crude anthocyanin was loaded, and this was repeated 10 times so that the same components were combined and concentrated to afford isolated anthocyanins.

For recycling preparative HPLC, two twin Waters Sunfire preparative columns (19 mm × 250 mm, 10 μm) were used and manually switched by a 10 port-2 position valve. The separations were performed with a STI 501 solvent pump (Saizhi, Hangzhou, China) with a UV-501 UV detector. 

**Figure 7:**
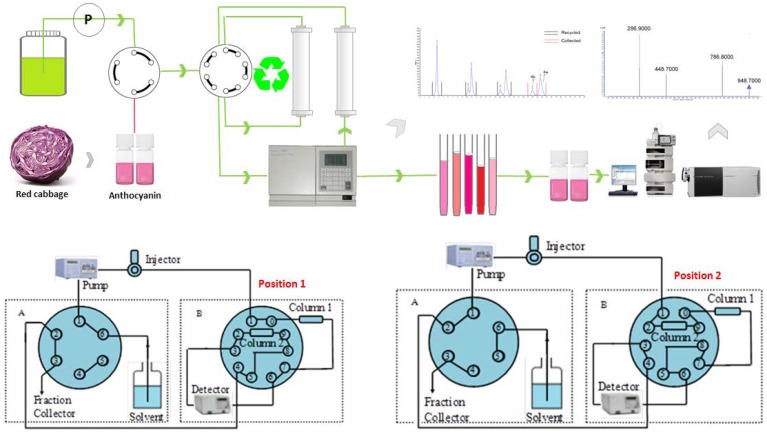
The schematic diagrams illustrating the extraction, separation, direct recycling, and alternate recycling systems.

Figure 7 shows the instrumental scheme in detail. For the process of recycling preparation, a sample solution was loaded by a manual injector and directed to the first column, and the separation of the objective analyte was monitored by the UV detector (Figure 7, position 1). After the sample injection, the 10-port valve was switched to position 2 to exclude unwanted low retention constituents. Once compounds of interest started to appear in the chromatogram, the 10-port valve was actuated again, and analytes were redirected to column 2. This procedure was repeated until sufficient separation was achieved. The resolved peaks were collected at position 2. All recycling preparations were performed using an isocratic elution, and thus, a preliminary screening for solvent composition was performed prior to the preparative separation.

### 4.5. The Structural Identification of Anthocyanin Monomers

The structural identification of isolated anthocyanin monomers was performed with high-performance liquid chromatography-diode array detector-tandem mass spectrometry (HPLC-DAD-MS/MS) analysis, and the results were compared with published mass data and their UV spectra. For MS/MS analysis, 2 μL of isolated monomers was injected separately, and the precursor ions were determined by a full scan mode with *m*/*z* from 100 to 1500. Once the precursor ions were determined, an automatic optimization process was performed to obtain the optimum conditions for product ion scan mode [24,25]. The MS/MS spectra and their UV spectra were used for structural elucidation.

### 4.6. The Photostabilities of Various Isolated Anthocyanin Monomers

The photodegradation of pure anthocyanin was evaluated using a previously reported protocol [6]. The methanol solutions of various anthocyanins were prepared with the same concentration. The solutions were then kept in the dark, under simulated solar light, and under natural indoor light, all at room temperature. The solutions were analyzed using HPLC to track the concentration change during the storage process. The stability of anthocyanins is highly dependent on light conditions, and thus, the photostability evaluation was performed using three different conditions: natural indoor light, darkness, and simulated solar light. The temperatures for the three different light conditions were at the same room temperature. The simulated solar light was provided by a simulated solar light irradiation cabinet using a 50 watt xenon lamp. All anthocyanin solutions were prepared in methanol with hydrochloric acid (0.01 M) at a 1 mg/mL concentration. The degradation of anthocyanins was determined with HPLC and compared to the peak areas. The freshly prepared anthocyanin solutions were used as 100%, and thus, the trend line of anthocyanins is highly related to their stability.

## 5. Conclusions

In the presented research, ten major anthocyanin monomers from red cabbage pigment extract were isolated by preparative and recycling preparative HPLC with purities of up to 99%. The photostability of anthocyanin monomers were investigated under various conditions. The results showed that the anthocyanins from red cabbage were mainly cyanidin derivatives with 3-diglucose and 5-glucose as aglycones and were acylated with various aromatic and aliphatic acids. The photostability of red cabbage anthocyanins monomers was significantly affected by the number of acylated groups. Anthocyanins with more acyl groups appeared more labile to photodegradation. Furthermore, recycling preparative chromatography demonstrated great potential for the efficient isolation of high purity anthocyanin monomers with similar polarities and structures, even for those not resolved by analytical high performance liquid chromatography.

## Figures and Tables

**Table 1:** Qualitative identification of anthocyanin monomers isolated from red cabbage anthocyanin

Peak	ts (min)	PDA	M (*m*/*z*)	Fragment Ions (*m*/*z*)	Identified Anthocyanin
1	8.936	510, 280	772.8	610.8, 488.8, 286.9	Cy-3-soph-5-Glc [24]
2	11.674	525, 330, 280	978.7	816.9, 449.0, 286.8	Cy-3(sin)-diGlc-5-Glc [25]
3a	15.928	525, 325, 280	1080.7	918.7, 448.8, 286.8	Cy-3-(caff-pC)-diGlc-5-Glc [26]
3b	16.283	525, NR	1110.6	948.8, 448.5, 280.7	Cy-3-(glucofer)-diGlc-5-Glc [27]
3c	16.607	525, NR	1140.8	978.6, 449.0, 286.8	Cy-3-(glucosin)-diGlc-5-Glc [26]
4a	21.853	525, 325, 280	918.9	765.9, 448.9, 286.8	Cy-3-(pC)-diGlc-5-Glc [28]
4b	22.046	525, NR	948.8	786.9, 448.7, 286.8	Cy-3-(fer)-diGlc-5-Glc [28]
5a	24.032	535, 320, 285	1124.8	963.0, 448.5, 286.8	Cy-3-(fer)(fer)-diGlc-5-Glc [24]
5b	24.189	535, NR	1155.1	992.7, 448.8, 286.7	Cy-3-(sin)(fer)-diGlc-5-Glc [29]
5c	24.458	535, NR	1184.8	1022.9, 448.8, 286.8	Cy-3-(sin)(sin)-diGlc-5-Glc [30]

Abbreviations: cyan: cyanidin, soph: sophoroside, Glc: glucoside, sin: sinapoyl, caf: caffeoyl, pC: p-coumaroyl, glucofer: glucopyranosyl-feruloyl, glucosin: glucopyransoyl-sinapoyl, fer: feruloyl. NR indicates that the PDA spectra were not resolved due to co-eluting compounds, and in these cases, the first PDA values represent the entire peak.
